# Endoscopic Treatment With Stenting, Cricopharyngeal Myotomy, and Vacuum-Assisted Closure of Iatrogenic Cervical Esophageal Perforation

**DOI:** 10.14309/crj.0000000000002157

**Published:** 2026-06-02

**Authors:** Yassmin Hegazy, Shou-jiang Tang, Parker Mullen, Jeremy Morgan, Jacob Moremen

**Affiliations:** 1 Division of Gastroenterology, Department of Medicine, University of Mississippi, Jackson, MS; 2 Division of Thoracic Surgery, Department of Surgery, University of Mississippi, Jackson, MS; 3 Division of Internal Medicine, Department of Medicine, University of Mississippi, Jackson, MS

**Keywords:** myotomy, stenting, perforation

## Abstract

Cervical esophageal perforations rarely require procedural intervention; however, in severe cases, surgical or endoscopic management should be considered. Our case highlights an endoscopic alternative for treatment using esophageal stenting, cricopharyngeal myotomy, and endoluminal vacuum-assisted closure for an unhealed iatrogenic case of cervical esophageal perforation.

## INTRODUCTION

Esophageal perforations are a rare but serious condition associated with significant morbidity and mortality with 25% of perforations occurring in the cervical esophagus.^1^ Patients with cervical esophageal perforations often heal spontaneously with conservative therapy and rarely require procedural intervention.^2^ In severe cases, traditional surgical management may be necessary including abscess drainage or primary repair of the defect.^3,4^ While esophageal stenting has provided an alternative to surgical treatment of esophageal perforations, it is considered an ineffective treatment of perforation at or above the cricopharyngeus given patient intolerance secondary to discomfort and foreign body sensation, increased risk of stent migration into the hypopharynx, and pulmonary aspiration.^5–7^

In addition to esophageal stenting, negative pressure wound treatment or endoluminal vacuum-assisted closure (EVAC) can be used as primary treatment of esophageal perforations and subsequent abscesses allowing continuous drainage of the infected fluid while promoting granulation tissue formation.^8–10^ Cricopharyngeal (CP) peroral endoscopic myotomy has been used to treat dysphagia caused from the CP bar with limited data in its use in esophageal perforations to allow for improvement in visualization while relieving the high-pressure zone near the upper esophageal sphincter to facilitate healing.^11,12^ Our case highlights the use of a combination of several endoscopic interventions to treat an iatrogenic case of cervical esophageal perforation using CP myotomy, esophageal stenting, and EVAC.

## CASE REPORT

A 76-year-old woman with a history of hypertension and diabetes presented for an index endoscopic ultrasound (EUS) for evaluation of nausea, vomiting, and left upper quadrant pain after computed tomography imaging showed a soft tissue mass along the lesser curvature of the proximal stomach.

**Figure 1.**
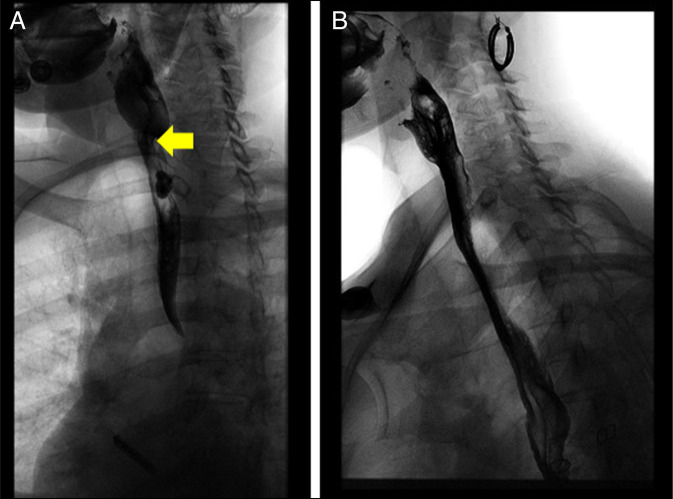
(A) Initial barium esophagram showing submucosal dissection and contrast leak. (B) Barium esophagram 1 month postesophageal perforation with no contrast leak of perforation.

During EUS, the patient sustained an iatrogenic submucosal dissection leading to a contained mediastinal perforation. computed tomography imaging and esophagram showed an extraluminal injury extending from the cervical to the distal thoracic esophagus (Figure 1). Repeat esophagram showed persistent contrast leak into the cavity after 5 days of conservative management with intravenous antibiotics and nothing by mouth. On admission, the patient met systemic inflammatory response syndrome criteria with a heart rate of 102 beats/minute, leukocytosis with a white blood cell count of 13.3, and a temperature of 99.6°F. She was started on intravenous antibiotics with piperacillin/tazobactam and empiric fungal coverage with intravenous fluconazole on admission and continued for a total of 16 days. She was initially admitted to the general medicine service and transferred to the thoracic surgery primary service. She remained on the floor given stable hemodynamics without the need for continued postoperative intubation or pressor requirements.

**Figure 2.**
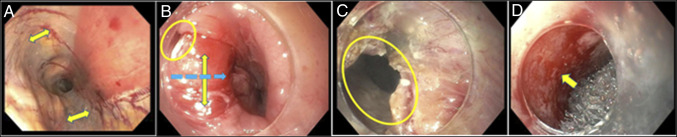
(A) Endoscopic ultrasound with submucosal dissection (arrows). (B) Cricopharyngeal bar (yellow arrow), perforation (circle), and myotomy pathway (blue arrow). (C) Defect following cricopharyngeal myotomy (circle). (D) Granulation tissue (arrow) following endoluminal vacuum-assisted closure sponge.

Given that initial EUS findings were concerning for a submucosal tunnel later judged to be a contained mediastinal perforation on repeat esophagram, the patient underwent an endoscopy which revealed a perforation at the cricopharyngeus with a long tunnel (Figure 2) not amenable to clip or suture closure. A prominent CP bar was present (Figure 2) likely as an instigating factor and obstructing our instrumentation of the defect.

Using standard tunneling technique, a full CP myotomy was performed giving greater access to the nonhealing wound (Figure 2). Mucopurulent fluid was lavaged from the cavity, and an overtube was placed into the defect to facilitate EVAC placement. A granulofoam sponge was sutured to a Blake drain and pushed into the cavity then brought out through the nose and secured to the vacuum-assisted closure cannister. She underwent 2 EVAC placements over 10 days until the wound was clean and sufficient granulation present (Figure 2). A 23 × 120 mm fully covered stent was placed just below the esophageal inlet (18 cm from the incisors) to collapse the cavity and accelerate closure of the now clean defect, beginning 15 cm from the incisors. A percutaneous endoscopic gastrostomy tube was used for nutrition throughout treatment. The stent was removed 39 days post-EUS, 2 weeks after placement. Repeat esophagram showed no esophageal perforation or leak (Figure 1). The patient was seen in clinic 1-month postdischarge with resolved dysphagia and tolerating oral intake.

## DISCUSSION

Cervical esophageal perforations that do not heal spontaneously are rare and often difficult to manage given the anatomy of the upper esophagus. The narrowed lumen and high-pressure zone from the cricopharyngeus limits space for additional endoscopic instrumentation while contributing to mechanical stress that can limit healing. In addition, the risk of migration to the hypopharynx, aspiration, and foreign body sensation causing patient intolerance makes stenting alone not an ideal option.

Our case demonstrates a multimodal endoscopic intervention that can address the anatomic difficulty in the treatment of cervical esophageal perforations. Using endoscopic myotomy of the CP bar improves visualization and access for ongoing instrumentation. This was augmented by EVAC, which provided continuous management of infection by allowing for drainage of mucopurulent fluid and promoting granulation formation at the perforation site. The covered esophageal stent helped collapse the cavity and allowed for accelerated healing of the clean defect following EVAC therapy. This type of intervention can be implemented and only available at centers of excellence with experienced endoscopists.

While surgical management can be considered for nonhealing esophageal perforations, endoscopic intervention can be used as a minimally invasive alternative. Our case demonstrates that a multimodal endoscopic intervention with CP myotomy, esophageal stenting, and EVAC can be successful in treatment of esophageal perforation at experienced centers and should be considered in patients with medical comorbidities.

## DISCLOSURES

Author contributions: Y. Hegazy, S. Tang, and J. Moremen: data collection, writing of manuscript. P. Mullen: data collection. J. Morgan: review of manuscript. J. Moremen is the article guarantor.

Previous presentation: This case was presented at the ACG Annual Scientific Meeting, October 27, 2025; Phoenix, Arizona.

Financial disclosure: None to report.

Informed consent was obtained for this case report.

## ABBREVIATIONS:

CP: Cricopharyngeal
EUS: Endoscopic Ultrasound
EVAC: Endoluminal vacuum-assisted closure
